# Social Media in Physician Education

**DOI:** 10.7759/cureus.19081

**Published:** 2021-10-27

**Authors:** Yingyot Arora, Noah Llaneras, Nyanika Arora, Roger Carillo

**Affiliations:** 1 School of Medicine, University of Miami Miller School of Medicine, Miami, USA; 2 Medicine, Florida International University, Herbert Wertheim College of Medicine, Miami, USA; 3 Biological Sciences, University of Denver, Denver, USA; 4 Cardiovascular Surgery, Palmetto General Hospital, Hialeah, USA

**Keywords:** social media communication, cardiac electrophysiology, online medical education, guidelines in medicine, pacemaker lead fracture, pacemaker lead infection, pacemaker lead extraction

## Abstract

Background

Social media has fundamentally changed the practice of medicine. It has taken the medical community by storm, benefited patient care, and has become a leading source for distributing medical information. Social media platforms are a low-cost, low-barrier entry means for health systems to highlight their competitive advantage to patients and providers alike. This study aimed to assess the role of social media in the education of physicians.

Methods

To evaluate the utility of social media in engaging physicians, four vignettes were utilized to highlight Class 1 indications for transvenous lead extraction (TLE), an electrophysiology procedure aimed at removing infected or damaged cardiac device leads. Individuals, via Twitter, were presented with cases and multiple-choice response options to determine the next best step in management.

Results

The clinical vignettes were seen by more than 18,000 individuals worldwide. Survey results indicated that 83% of individuals who participated had correctly identified class 1 indications for this life-saving procedure. Sixteen percent to 21% of physicians incorrectly identified the next step in the management of Class 1 indications for lead extraction, representing a need for education and an opportunity to inform and educate.

Conclusion

Social media may be a useful tool in physician education. However, guidelines and further research are needed to continue to understand the role of social media within the medical field.

## Introduction

Two-hundred-eighty characters can change the world. Twitter has revolutionized the way information is disseminated. A clever hashtag paired with a well-crafted message can inform, educate, and influence in a way that puts the most prestigious articles to shame. Social media platforms, such as Twitter, are internet-based tools that allow users to create, share, and participate in content as part of a social network. These networks have the unique ability to connect people from around the world. Physicians have harnessed this tool to influence practice guidelines and promote quality patient care [1]. Medical professionals have used Twitter to increase exposure to new research, network with experts, and improve patient communication. For example, interventional cardiologists have utilized Twitter to establish radial access as the gold standard for cardiac catheterization [2]. Electrophysiologists have also displayed the importance of using Twitter to resurrect HIS bundle pacing (HBP) as a viable pacing technique. The #dontdisthehis movement has become so popular that the Heart Rhythm Society (HRS) has utilized it in webinars and virtual sessions [3]. It is vital that experts in a field adequately engage online to control the narrative regarding safety, efficacy, and indications for their procedures. If not, inaccurate information circulates quickly, and patients will suffer as a result. Our study sought to investigate the impact of social media on continuous physician education.

## Materials and methods

All surgical electrophysiology cases from our single tertiary care center in Miami, Florida, performed between January 2018 and April 2020, were evaluated on a threefold criterion: 1. Relevance to physicians in both the lead extraction and the greater cardiology community, 2. Illustration of extraction guidelines set forth by the Heart Rhythm Society (HRS) lead extraction guidelines [4], 3. Patient consent and willingness to engage. Four cases that met the criteria were selected and condensed into 140-character clinical vignettes that were posted on Twitter. Each vignette provided pertinent case details (demographics, lab values, physical exam, and medical history) and associated imaging. Participants, who were mostly electrophysiologists connected with our senior author on Twitter, voted on polls and asked clarifying questions in comment replies. Polls were open for 24 hours, during which participants could cast their vote for the appropriate next best step in management. When appropriate, we provided additional case information in subsequent tweets. After the 24 hours had elapsed and the polls ended, the correct answer was revealed. Our chief researcher subsequently discussed a review of management guidelines. Patient status updates were shared when appropriate. Response and engagement data were collected and processed through Twitter's native data analytics and insights platform. Results of the 24-hour Twitter poll and the number of times the tweets were viewed in the 30-day period following the case presentation were recorded for each case.

**Figure 1:**
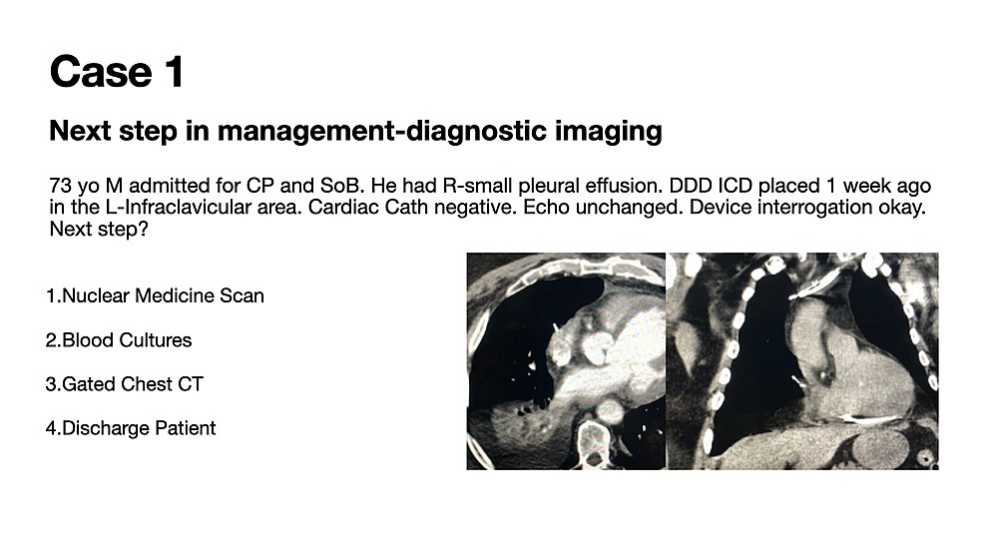
Case 1: Appropriate diagnostic imaging for suspected lead perforation

Case 1 details the appropriate diagnostic imaging for suspected lead perforation (Figure 1).

**Figure 2:**
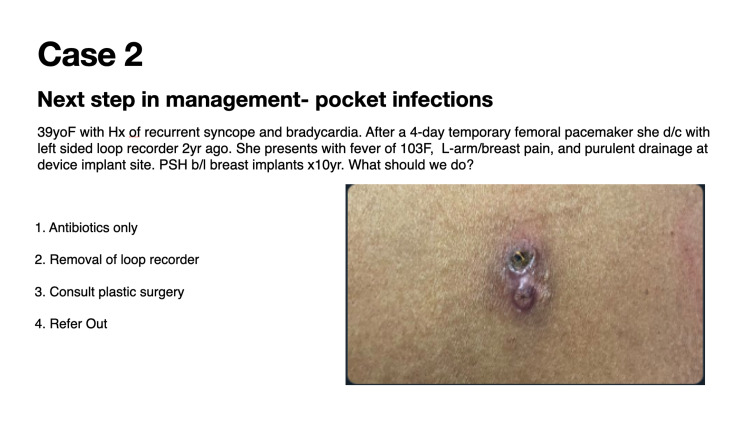
Case 2: Next step in the management of pocket infections

Case 2 details the next step in the management of pocket infections (Figure 2).

**Figure 3:**
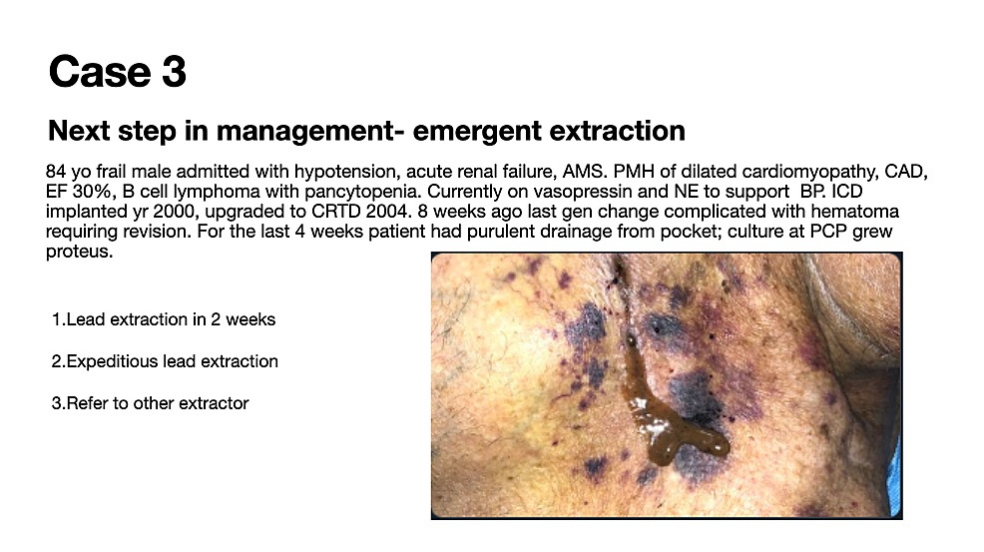
Case 3: Indications for emergent lead extraction

Case 3 evaluates indications for emergent lead extraction (Figure 3).

**Figure 4:**
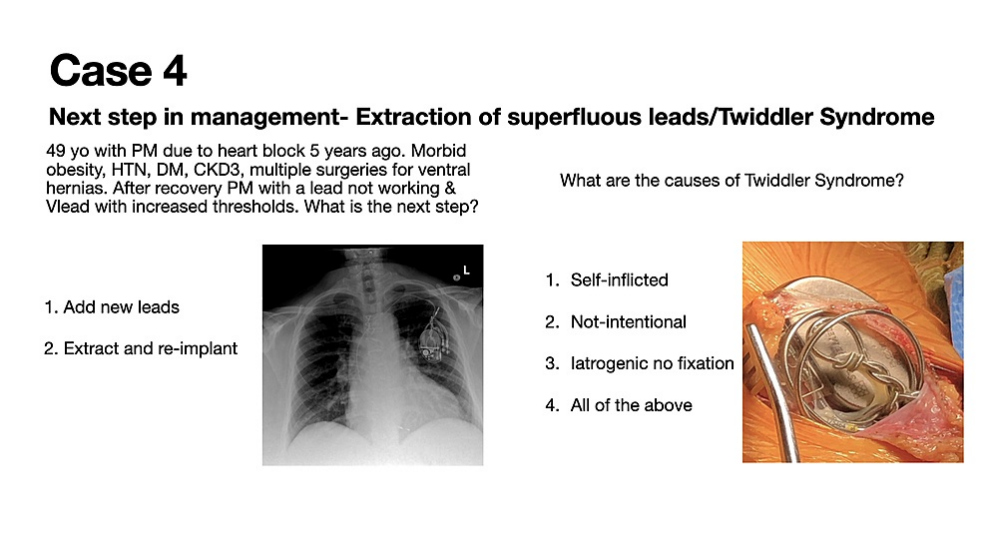
Case 4: Extraction of superfluous leads and management of Twiddler Syndrome

Case 4 describes a case of extraction of superfluous leads and the management of Twiddler Syndrome (Figure 4).

## Results

Case 1

**Figure 5:**
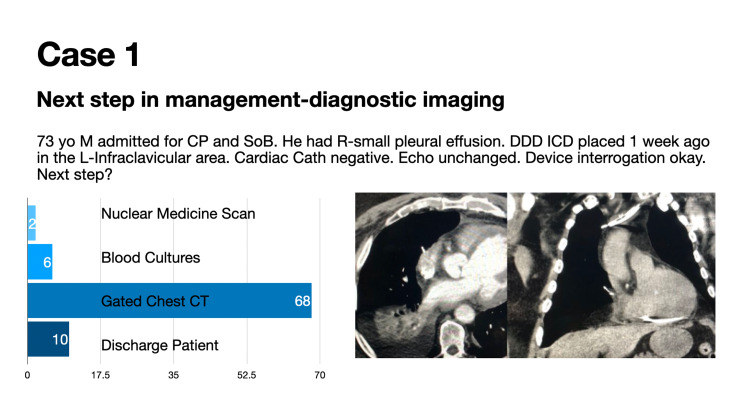
Case 1 results

Eighty-four (84) participants voted in this poll during a 24-hour timeframe. This vignette was seen by over 6500 individuals in the 30-day period following the presentation of the case. Sixty-eight (68; 79%) of 84 poll participants correctly identified that a gated chest CT is the appropriate imaging modality for identifying ventricular lead perforation (Figure 5). HRS best practice guidelines suggest that electrocardiogram graded cardiac CT appears to be more accurate than chest radiography to diagnose lead perforation [4].

Case 2

**Figure 6:**
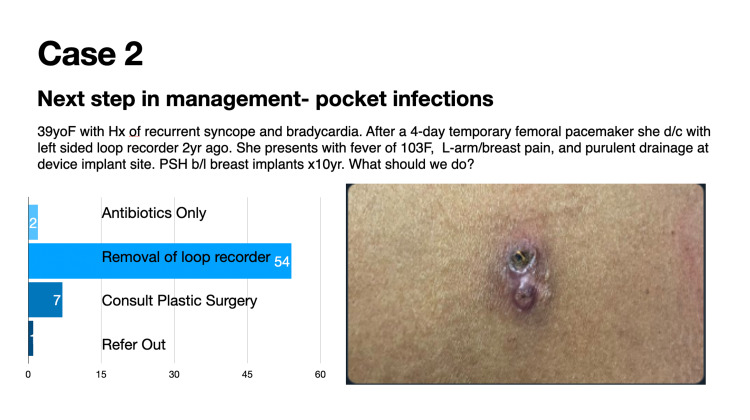
Case 2 results

Forty-four (44) participants voted in this poll during a 24-hour timeframe. This vignette was seen by over 5000 individuals in the 30-day period following the presentation of the case. Fifty-four (54; 84%) of 64 poll participants correctly identified that device removal is the appropriate next step in management in case of an infected device (Figure 6). Device infection is a class 1 indication for lead extraction. The entire explanted leads or lead tips should be sent for culture, as the sensitivity of tissue culture is higher than that of swab cultures of the pocket [4]. However, it is crucial to consider the potential for lead contamination when leads are extracted through the generator pocket.

Case 3

**Figure 7:**
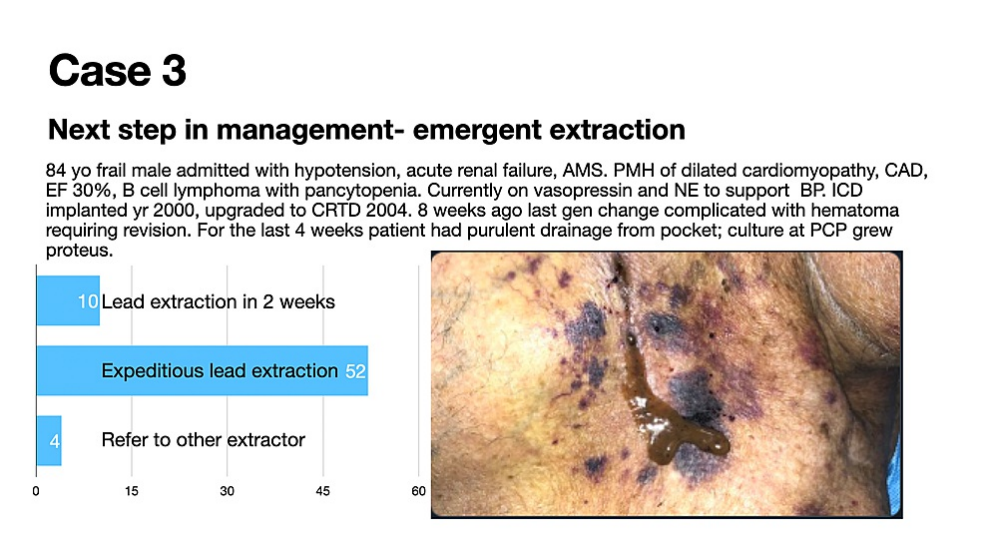
Case 3 results

Sixty-six (66) participants voted in this poll during a 24-hour timeframe. This vignette was seen by over 5200 individuals in the 30-day period following the presentation of the case. Fifty-two (52; 79%) of 66 poll participants correctly identified that device removal is the appropriate next step in management to manage an infected device with purulent drainage (Figure 7). If antibiotics are going to be prescribed, guidelines suggest that at least two blood cultures are obtained before starting antibiotic therapy [4].

Case 4

**Figure 8:**
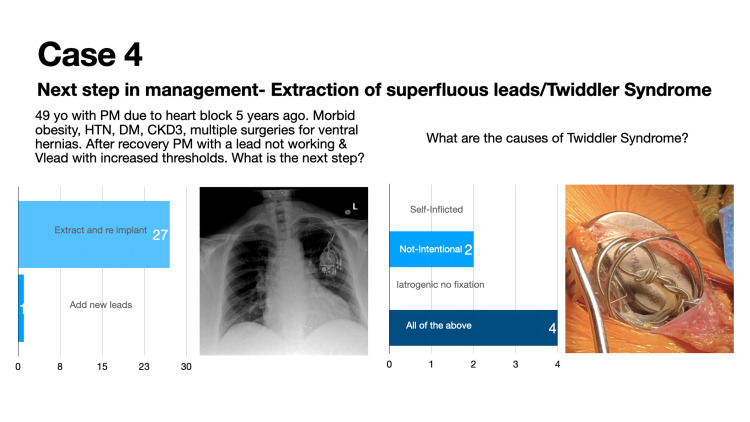
Case 4 results

Twenty-eight (28) participants voted in this poll during a 24-hour timeframe. This vignette was seen by approximately 1300 individuals in the 30-day period following the presentation of the case. Twenty-seven (27; 96%) of 28 poll participants correctly identified that device extraction and reimplantation is the appropriate next step in the management of suspected Twiddler Syndrome. Four (4; 60%) of six poll participants correctly identified that Twiddler Syndrome can be self-induced or iatrogenic secondary to improper lead fixation (Figure 8). Twiddler Syndrome is a rare cause of pacemaker malfunction that occurs when a patient inadvertently or deliberately manipulates the generator within the pocket. This results in coiling of the lead and failure of ventricular pacing [5].

We analyzed the Twitter profiles of all participants engaging in the comments section of our four vignettes. All of the tweets and subsequent interactions in the comments were from individuals who self-identified as physicians on their Twitter handles. We extrapolated these results to deduce that a majority, if not all, of the individuals voting on our polls were physicians.

## Discussion

Social media has already proven to be effective in sparking global revolutions in changing practice guidelines among cardiologists. Social media played a role in the resurgence of interest in HBP - a technique first proposed in the 1970s that gained traction 40 years later, in 2013-2014, when two American physicians began tweeting cases with the hashtag "#dontdisthehis" [3,6]. This hashtag sparked a movement among cardiologists worldwide to share issues, discuss their own experiences, and seek advice from peers worldwide through a series of rapid 140-character exchanges from the palms of their hands. Twitter played a pivotal role in exposing young cardiologists to a pacing technique that lived in relative obscurity for almost 40 years. It allowed experienced physicians to share their insights and collaborate in a way that had previously been restricted to conferences. Now, thanks to #dontdisthehis, HBP is a staple of electrophysiology departments nationwide. The Twitter #radialfirst movement was also a remarkable display of the power social media has to engage physicians and influence evidence-based guidelines. A series of tweets catalyzed a global movement to promote radial artery used as a superior access technique for cardiac catheterization. Hundreds of physicians globally engaged in discourse to discuss the merits of radial access for cardiac catheterization, and the radial artery has now generally become the preferred access technique [2].

While social media use is prevalent among other cardiology subspecialties, its use by the lead extraction community is low. It is estimated that approximately 90% of doctors and over 93% of medical students in the United States are active on social media [2]. A more robust online presence may be valuable in sharing best-practice guidelines and reducing patient care delays. Our case studies demonstrate that an urgent call to action is needed. Up to 16-21% of physician respondents did not identify the Class 1 indication for extraction in these critical cases [4]. Delayed intervention can result in considerable morbidity and mortality for patients with cardiovascular implantable electronic device (CIED) infections [7]. Developing effective means for preventing device infection and early diagnosis is vital in decreasing the mortality, morbidity, and medical cost related to lead infections. Social media can be leveraged as a low-cost, low barrier of entry to address these barriers to care.

During a national conference on lead extraction, we shared our preliminary study findings and motivated further clinical research. This led to the creation of the “No Infection Left Behind” task force to address cardiac device infections. Our data suggested that these infections may be underdiagnosed and mismanaged. Based on these results, a group of experienced lead extractors from Duke University conducted a follow-up analysis of a 2017 retrospective review of Medicare data on device infections [8-9]. This analysis was the first of many clinical research projects that stemmed from our presence online. Additionally, we collaborated with industry colleagues to initiate a physician education campaign to highlight the benefits and safety of lead extraction. Case reports of life-threatening complications from first-generation extractions overshadow recent data showing the safety and efficacy of this procedure [9-10]. This falsely elevated perception of risk is exacerbated by the presence of vocal critics on social media. Meanwhile, literature shows that extractions are often the best management approach for most patients [9-10]. Social media played a crucial role in identifying and addressing these lapses in patient care.

A symbiotic relationship between extractors and health systems can significantly benefit physicians, healthcare facilities, and patients. Seventy percent of U.S. health care organizations are active on social media [1]. In an increasingly competitive environment, health systems must utilize all tools available to distinguish themselves to create a competitive edge. A recent study found that 57% of consumers reported that a hospital's social media presence would strongly influence their healthcare decisions. Ninety percent of respondents 18-24 years of age disclosed that they trust medical information shared by health professionals on their social media networks [1]. By developing a robust online presence, extractors can highlight the competitive advantage of their institutions while creating an influential professional and patient network.

Healthcare professionals can utilize social media to gain exposure to individuals seeking out healthcare for the first time. Twenty-year-olds are more than twice as likely as fifty-year-olds to use social media for health-related discussions [11]. Engaging online is critical to prevent healthcare professionals and medical centers from losing the attention of future generations. Ninety percent (90%) of respondents 18-24 years of age disclosed that they trust medical information shared by health professionals on their social media networks [11]. Thirty-one percent (31%) of healthcare professionals use social media for professional networking [1,12]. Patients are likely to share their medical experience online via social media with other patients. Medical centers can focus on this to improve patient-doctor interaction in the future. Successful cardiologists have created social media profiles catering to patients and connecting them to global resources [13]. Studies have shown that the additional virtual touchpoint can help patients feel more engaged and connected with their physicians, leading to higher satisfaction and engagement in care [12].

Social media has also been invaluable for professional societies to modernize their approaches to provider outreach and engagement. In a world of constant data overload, essential updates to best practices may easily get lost in the background. Several specialties have developed unique approaches to address this ever-growing concern. Emergency Medicine and Nephrology are two fields where social and interactive media have transformed the concept of lifelong learning and continuous education. The Annals of Emergency Medicine partnered with Academic Life in Emergency Medicine to create an educational and wellness-based blog to create a novel approach to accelerating knowledge transition in the digital era [14]. Meanwhile, nephrologists have utilized games, online journal clubs, and digital mentorship platforms to provide a unique array of engaging educational experiences that physicians can access 24 hours a day [1,15]. By dispersing learning points into interactive, easy-to-digest bits of information, these professional societies have modernized how information is shared.

If not utilized correctly, social media can lead to significant harm to both providers and patients alike. Some disadvantages include: 1) The quality and reliability of social media are poor. Most posts are not subjected to proofreading and can contain misleading and false information. 2) Partaking in social media can be time-consuming. 3) Social media has the risk of damaging the professional image. 4) The utmost level of caution must be exercised to protect patient privacy. The ethical considerations of discussing, and potentially impacting, health-related policy issues or clinical guidelines on social media platforms cannot be taken lightly. Despite these very real dangers of social media, its role in healthcare is already established. Therefore, the lead extractors should leverage social media to benefit the community while prudently maintaining a professional reputation.

Limitations

There are several limitations to our study: we were not able to gather insight on the specific title or qualification of individuals who responded to our polls or the viewers of our Tweets. This has the potential to bias our results. Additionally, we presented a limited number of curated vignettes, which may have exposed us to a certain degree of responder bias. Both practical and ethical guidelines, as well as further research, are needed to understand better how electrophysiologists can continue to leverage social media in the future.

## Conclusions

Our study demonstrates that social media has effectively identified gaps in physicians' education and can be beneficial in ensuring adherence to best practice guidelines. We identified that up to 20% of experts in the field are potentially not following evidence-based guidelines by leveraging Twitter polls. Our series of tweets reached 18,000 people and facilitated physician education online. Additionally, we found that social media can foster collaboration and help pioneer new research in clinical medicine. Further research into establishing ethical guidelines for utilizing social media as a tool for shaping clinical practice is required.
